# Expression of *Vitreoscilla* hemoglobin enhances production of arachidonic acid and lipids in *Mortierella alpina*

**DOI:** 10.1186/s12896-017-0388-8

**Published:** 2017-08-30

**Authors:** Huidan Zhang, Yingang Feng, Qiu Cui, Xiaojin Song

**Affiliations:** 1 Shandong Provincial Key Laboratory of Energy Genetics, Qingdao Institute of Bioenergy and Bioprocess Technology, Chinese Academy of Sciences, No.189 Songling Road, Laoshan District, Qingdao, Shandong Province 266101 China; 2 Key Laboratory of Biofuels, Qingdao Institute of Bioenergy and Bioprocess Technology, Chinese Academy of Sciences, Qingdao, Shandong 266101 China; 3 Qingdao Engineering Laboratory of Single Cell Oil, Qingdao, Shandong 266101 China; 4 University of Chinese Academy of Sciences, Beijing, 100049 China

**Keywords:** Arachidonic acid, *Mortierella Alpina*, Hemoglobin, Dissolved oxygen, Fermentation

## Abstract

**Background:**

Arachidonic acid (ARA, C20:4, n-6), which belongs to the omega-6 series of polyunsaturated fatty acids and has a variety of biological activities, is commercially produced in *Mortierella alpina*. Dissolved oxygen or oxygen utilization efficiency is a critical factor for *Mortierella alpina* growth and arachidonic acid production in large-scale fermentation. Overexpression of the *Vitreoscilla* hemoglobin gene is thought to significantly increase the oxygen utilization efficiency of the cells.

**Results:**

An optimized *Vitreoscilla* hemoglobin (VHb) gene was introduced into *Mortierella alpina* via *Agrobacterium tumefaciens*-mediated transformation. Compared with the parent strain, the VHb-expressing strain, termed VHb-20, grew faster under both limiting and non-limiting oxygen conditions and exhibited dramatic changes in cell morphology. Furthermore, VHb-20 produced 4- and 8-fold higher total lipid and ARA yields than those of the wild-type strain under a microaerobic environment. Furthermore, ARA production of VHb-20 was also 1.6-fold higher than that of the wild type under normal conditions. The results demonstrated that DO utilization was significantly increased by expressing the VHb gene in *Mortierella alpina*.

**Conclusions:**

The expression of VHb enhances ARA and lipid production under both lower and normal dissolved oxygen conditions. This study provides a novel strategy and an engineered strain for the cost-efficient production of ARA.

**Electronic supplementary material:**

The online version of this article (doi:10.1186/s12896-017-0388-8) contains supplementary material, which is available to authorized users.

## Background

Arachidonic acid (ARA, 5,8,11,14-cis-eicosatetraenoic acid), belonging to the omega-6 series of polyunsaturated fatty acids (PUFAs), is not only a structural component of the cell membrane but also the biogenetic precursor of prostaglandins, leukotrienes, thromboxanes, and other eicosanoid hormones [1, 2]. Furthermore, ARA is widely used in health food, pharmacology, agriculture, cosmetics and other industries [3, 4]. *Mortierella alpina*, which has a high ARA content, is considered one of the best ARA-producing strains and has been used in industrial applications [5].

The growth of *M. alpina* is closely related to the dissolved oxygen, especially in high cell density fermentation. The fast growth of the mycelium can result in an apparent increase in broth viscosity. The mycelium can agglomerate easily and further decrease the efficiency of oxygen mass transfer [6, 7]. Therefore, improving the concentration of dissolved oxygen in the fermentation process or increasing the utilization of dissolved oxygen is key to improving the yield of ARA.

At present, there are various methods to improve the oxygen supply, for example, by increasing the stirring speed or ventilation, or by adding surfactants to increase the concentration of dissolved oxygen in the fermentation. However, these methods require either special equipment or high energy consumption [8, 9]. It has been shown that the expression of the bacterial (*Vitreoscilla*) hemoglobin (VHb) is an effective method to solve the problem of oxygen supply in the fermentation process because VHb can improve the efficiency of intracellular oxygen transport [10, 11]. VHb is an oxygen-binding protein produced by *Vitreoscilla*, which allows the *Vitreoscilla* (aerobic bacteria) to survive under limited oxygen conditions. Khosla and Bailey first cloned the *vgb* gene and successfully expressed it in *E. coli* [12].They demonstrated that the expression of VHb could promote cell growth, increase protein synthesis capacity under limited oxygen conditions [13, 14]. According to the characteristics of VHb, it has a good application, which has been successfully engineered into different bacteria, fungi, as well as some plants and animals, to improve cell growth and to increase the expression of exogenous protein and metabolite production [15, 16].

The metabolic engineering method also has been applied to improve the ARA production. For example, Hao et al. [17] studied overexpression of ME2 and G6PD2 in *M. alpina*, increasing the supply of intracellular NADPH, with a 1.7-fold increase in total fatty acid and a 1.5-fold increase in arachidonic acid (ARA) content. In 2005, Takeno et al. [18] improved of the fatty acid composition of *M. alpina* 1S-4, through RNA interference with Δ12 desaturase gene expression. But ARA biosynthesis is multi-step, subject to multiple factors. The modification of one or two genes is not easy to produce transformants with the desired trait. Overexpression of VHb could increase the intensity of respiratory chain and increase the supply of intracellular ATP, which may affect the expression of intracellular multi-gene expression and promote the formation of ARA.

To the best of our knowledge, VHb has not been used in *M. alpina* to date. In this study, we investigate the effect of VHb on growth and ARA production in *Mortierella alpina* and explored the mechanism of VHb in ARA synthesis.

## Methods

### Strains, plasmids and growth conditions

Wild-type *Mortierella alpina* ATCC 32222 was obtained from the American Type Culture Collection (ATCC, Manassas, VA, USA). The *Agrobacterium tumefaciens* strain C58C1 and plasmid pBIG4MRHrev were gifts from Yasuyuki Kubo (Kyoto Prefectural University, Japan). The strains, plasmids and primers used in this study are listed in Additional file 1: Table S1 and Table S2, respectively. The composition of the LB-Mg agar, minimal medium (MM) and inducing medium (IM), which were used for the culture, transformation and infection of *Agrobacterium tumefaciens*, respectively, have been described previously [19]. GY medium (20 g L^−1^ glucose, 10 g L^−1^yeast extract and 20 g L^−1^agar) was used for the transformation and screening of *Mortierella alpina*. *M. alpina* strains were grown on PDA medium (potatoes 200 g L^−1^, glucose 20 g L^−1^, and agar 20 g L^−1^) slants at 25 °C. The seed culture medium contained 30 g L^−1^ glucose, 6 g L^−1^ yeast extract, 3 g L^−1^ KH_2_PO_4,_ 2.8 g L^−1^ NaNO_3_, and 0.5 g L^−1^ MgSO_4_•7H_2_O. The medium for shake-flask culture contained 80 g L^−1^ glucose, 11 g L^−1^ yeast extract, 3.8 g L^−1^ KH_2_PO_4_, 3.5 g L^−1^ NaNO_3_, and 0.5 g L^−1^ MgSO_4_•7H_2_O. The medium for bioreactor fermentation contained 30 g L^−1^ glucose, 20 g L^−1^ yeast extract, 4 g L^−1^ KH_2_PO_4_, 3.8 g L^−1^ NaNO_3_, and 0.6 g L^−1^ MgSO_4_•7H_2_O. The *Escherichia coli* strains were grown in LB medium (10 g L^−1^ tryptone, 5 g L^−1^ yeast extract, and 10 g L^−1^ NaCl) at 37 °C.

### Codon analysis and optimization

The codon preference table was found by analyzing the codon preference of the gene of *M. alpina* by the online software Codon Usage Database (http://www.kazusa.or.jp/codon/). The *vgb* gene (GenBank accession no.M30794.1) of *Vitreoscilla* was optimized by software Optimizer (http://genomes.urv.cat/OPTIMIZER/) based on the codon preference table. The codon-optimized nucleotide sequence of *vgb* was synthesized by GenScript (Nanjing) Co., Ltd. (Jiangsu, China).

### Vector construction

The hygromycinB phosphotransferase gene (HPH) expression cassette containing the hisH4.1 promoter, the *hpt* resistance gene and the trpC terminator was amplified with primer pair P1/T1 from the vector pD4 [20]. This HPH expression cassette was inserted into pMD19-T to yield the plasmid pMD19T-HPH. The 18S sequence, which was amplified from *M. alpina* ATCC 32222 cDNA with primer pair 18S-F/18S-R, was ligated to the pMD19T-HPH vector, and the resulting construct was named pMD19T-HPH-18S. The carboxin resistance gene (CBXB) [21] was amplified with the primer pair CBXB-F / CBXB-R and used to replace the *hpt* gene, thus forming plasmid pMD19T-CBXB-18S. The CBXB expression cassette was amplified from plasmid pMD19T-CBXB-18S with primer pair P2/T2, digested with restriction endonucleases *Cla*I and *Eco*RI, and then ligated to T-DNA binary vector pBIG4MRHrev. The resulting plasmid was named pBIG-CBXB. The HisH4.1 promoter and trpC terminator were first amplified with primer sets P3-F/P3-R and T4-F/T4-R from the vector pBIG-CBXB, thus yielding fragments of 1.1 kb and 0.7 kb, respectively.

The optimized *vgb* sequence was inserted into pUC57 to create the plasmid pUC57-vgb (Additional file 1: Table S1). The primer pair VHb-F/VHb-R was used to amplify the optimized *vgb* gene from the vector pUC57-vgb, forming a 441 bp fragment. The *vgb* gene cassette containing the hisH4.1 promoter, the *vgb* gene and the trpC terminator was assembled by overlap PCR. The obtained 2.1 kb *vgb* gene cassette was digested with *Eco*RI and *Sma*I and then ligated to pBIG-CBXB, thus forming the plasmid pBIG-CBXB-VHb (Additional file 1: Figure S2).

### *Agrobacterium tumefaciens*-mediated transformation of *M. alpina*

The transformation method was optimized on the basis of previously described *Agrobacterium tumefaciens-*mediated transformation (ATMT) methods [19, 22]. *M. alpina* spores were collected from cultures growing on PDA agar medium, and the suspension was filtered through three layers of lens paper. *A. tumefaciens* C58C1 was electrotransformed with the plasmid pBIG-CBXB-VHb. The positive transformants were confirmed by PCR, and then a single colony containing plasmid pBIG-CBXB-VHb was selected to grow overnight in 5 mL of liquid LB-Mg medium containing 50 μg mL^−1^ kanamycin and 50 μg mL^−1^ rifampin at 28 °C and 200 rpm. The bacterial cells were collected by centrifugation at 5000 rpm for 3 min at room temperature and washed once with fresh IM, and then fresh IM was used to adjust the OD to 0.2-0.3. After being incubated at 28 °C until an OD660 of 0.8 was reached, 100 μL of the *Agrobacterium* culture was mixed with 100 μL of *M. alpina* spore suspension (10^8^ mL^−1^) and then spread onto cellophane membranes, placed on co-cultivation medium (IM with 1.5% agar) and incubated at 23 °C for 2 days. After co-cultivation, the cellophane membranes were transferred to GY plates containing 150 μg mL^−1^ carboxin, 50 μg mL^−1^ spectinomycin and 50 μg mL^−1^ cefotaxime to inhibit the growth of *Agrobacterium*. The plates were incubated at 25 °C until CBXB-resistant colonies became visible. The transformed candidates were transferred to GY agar plates containing 150 μg mL^−1^ carboxin. To obtain stable transformants, this procedure was repeated three times, and all experiments were conducted in triplicate.

### PCR verification of the transformants

PCR was used to identify whether the mycelium was successfully transformed. Genomic DNA of *M. alpina* strains was prepared by a method described previously [23]. The genomic DNAs of the transformants were used as PCR templates to confirm the integration of the plasmid pBIG-CBXB-VHb by using the *vgb* gene specific primers VHb-F and VHb-R (Additional file 1: Table S2). Wild type *M. alpina* and the plasmid pBIG-CBXB-VHb were used as negative and positive PCR controls, respectively. The PCR products were run on a 1.0% agarose gel and stained with ethidium bromide.

### Analytical methods for dry mycelial weight, total lipid and arachidonic acid production

#### Cell dry weight determination

In total, 50 mL cell suspensions were harvested by filtration at room temperature. The cell pellet was washed twice with distilled water and dried to constant weight at 50 °C. The dry cell weight was determined by weight analysis and represented by dry cell weight (DCW).

### Lipid extraction and fatty acid composition analysis

Fatty acid extraction and methylation were carried out according to the previously described methods [24]. Total lipids were extracted by using approximately 100 mg of mycelia (dry weight). Fatty acid methyl esters (FAMEs) were obtained by reacting the lipids at 85 °C for 2.5 h in the presence of 2% sulfuric acid/methanol (*v*/v). FAMEs were extracted in hexane and analyzed by gas chromatography (Agilent Technologies, 7890B) with an HP-INNOWAX capillary column (30 m by 0.25 mm, 0.25 μm film thickness). The oven temperature was set at 100 °C for 1 min, was raised to 250 °C at a rate of 15 °C per minute and then was held at 250 °C for 5 min. The split ratio was 1:19, and the carrier gas was nitrogen. The injection volume was 1 μL. Peak detection used a flame ionization detector (FID). The temperature of the flame ionization port and injection port was 280 °C.

### Shake-flask culture and bioreactor scale fermentation

To study the effect of VHb on *M. alpina*, especially under the oxygen-restricted conditions, we compared the DCW, total lipid content, and arachidonic acid production between the transformant VHb-20 and the wild-type strain in shake flasks and a bioreactor under dO_2_ limiting and non-limiting conditions. First, the VHb-20 transformants and wild type were inoculated into six 250 mL shake flasks with baffles. Three shake flasks contained 50 mL medium (normal, dO2 non-limiting condition), and three contained 150 mL medium (dO2 restriction). They were shaken at 25 °C and 200 rpm for 7 days. Meanwhile, bioreactor cultures were grown in 5-L Biostat B plus bioreactors (Sartorius Stedim Biotech, Germany) with 3 L of medium. The rate of ventilation and agitation during growth in the bioreactor was set to non-limiting oxygen (1.7 vvm, 200 rpm) and limiting oxygen (0.8 vvm, 100 rpm) conditions. The transformant and wild-type cultures were grown for 7 days at 25 °C, pH 6.5. The glucose concentration was maintained at approximately 20 g L^−1^ by feeding the 80% (w / v) glucose solution, which was measured with a SBA-40D Biosensor (Institute of Biology, Shandong Academy of Sciences, China). Twenty milliliter samples were taken at 24 h intervals in all experiments. The entire fermentation process used bioprocess-automated software for monitoring and control.

### Quantitative real-time PCR (qRT-PCR) validation

The expression levels of some desaturase genes, such as Δ9, Δ12, Δ6, and Δ5 desaturase, were further quantified by real-time PCR to validate the effects of VHb expression on the biosynthesis of unsaturated fatty acids. The primer sequences (Additional file 1: Table S3) were designed according to the genome sequencing data (NCBI BioProjects PRJNA41211). Total RNA was extracted using a thermo Scientific GeneJET RNA Purification kit (No. K0731). After DNA degradation by DNase, 2 μg of total RNA was reverse transcribed using a TIANScript reverse transcription Kit (Tiangen) in a 20 μl reaction volume.

qRT-PCR was performed in a LightCycler R480 Real-time Detection System (Roche). A melting curve analysis of the amplification products was performed to confirm the specificity of amplification. To quantify the transcription of each gene, the copy number was determined by generating a standard curve by using a serial 10-fold dilution of the targeted PCR product inserted into the pMD™ 19-T vector (TaKaRa Bio Group). For sample normalization, 18S rRNA was used as an internal standard. All the reactions were performed in triplicate, and the data were normalized by using the average values for the internal standard.

### Calculation and statistical analysis

All results are given as the mean ± standard error of the mean (SEM), and significant differences (*P* < 0.05, *P* < 0.01) between means were compared by the T-test.

## Results

### The stabilized expression of VHb in *M. alpina*

To express VHb efficiently in *M. alpina*, we designed and optimized the *vgb* gene according to the codon preference in *M. alpina.* The optimized *vgb* gene sequence is shown in Additional file 1: Figure S1.

We first tested the sensitivity of *M. alpina* ATCC 32222 to carboxin and another series of antibiotics (Additional file 1: Table S4). The strain did not grow in the presence of 150 μg mL^−1^ carboxin but showed high resistance to other antibiotics. Therefore, the carboxin resistance gene (*CBXB*) was used as the selection marker [21, 25] at a screening concentration of 150 μg mL^−1^.

**Fig. 1:**
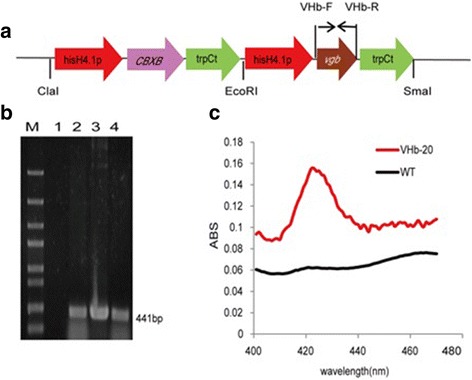
Transformant verification. **a** Scheme for expression of vgb in *Mortierella alpina* ATCC 32222; **b** PCR analysis of transformants of *Mortierella alpina*. The PCR product corresponding to VHb is 441 bp (Lanes: M, 5000 bp marker; 1, negative control, wild-type strain ATCC 32222; 2, transformant VHb-20; 3, positive control, plasmid pBIG-CBXB-VHb; 4, transformant VHb-10); **c** CO binding spectra of cell extracts from original strain ATCC 32222 and transformant VHb-20

The plasmid pBIG-CBXB-VHb was transformed into *M. alpina* ATCC 32222 by ATMT. All clones were verified by PCR using genomic DNA as templates. To select the high-yield ARA strain, 14 correct clones verified by PCR were randomly selected to check ARA production in limiting and non-limiting oxygen conditions. After 7 d of cultivation, a positive clone exhibiting the highest ARA production (VHb-20) was identified. Thus, VHb-20 strain was chosen for all further experiments. The *vgb* gene expression cassette inserted in clone VHb-20 was confirmed by genomic PCR amplification (see Fig. 1a and b).

To confirm the functional expression of VHb in VHb-20, the carbon monoxide (CO) difference spectra were measured, and the wild-type strain was used as a control. VHb-20 showed a typical VHb CO-binding absorption peak at 419 nm, whereas this absorption peak was not observed in the control (Fig. 1c). This result indicated that the VHb expressed in VHb-20 was biologically active. Afterwards, VHb-20 was continuously inoculated and cultivated on carboxin-free PDA solid medium, and the integrated *vgb* gene in the genome was monitored by PCR. The result indicated that the recombinant strain was genetically stable after 5 sub-cultivation cycles.

### Effects of VHb expression on the growth and ARA production of *M. alpina*

Both VHb-20 and the wild-type strain were cultivated in 250 mL shake flasks under limiting and non-limiting oxygen conditions, and their growth patterns and ARA production were investigated. The results showed that the VHb-20 strain grew faster than the wild-type strain and formed more homogeneous mycelial pellets under both conditions. In addition, the hyphae of *M. alpina* ATCC 32222 were looser.

**Fig. 2:**
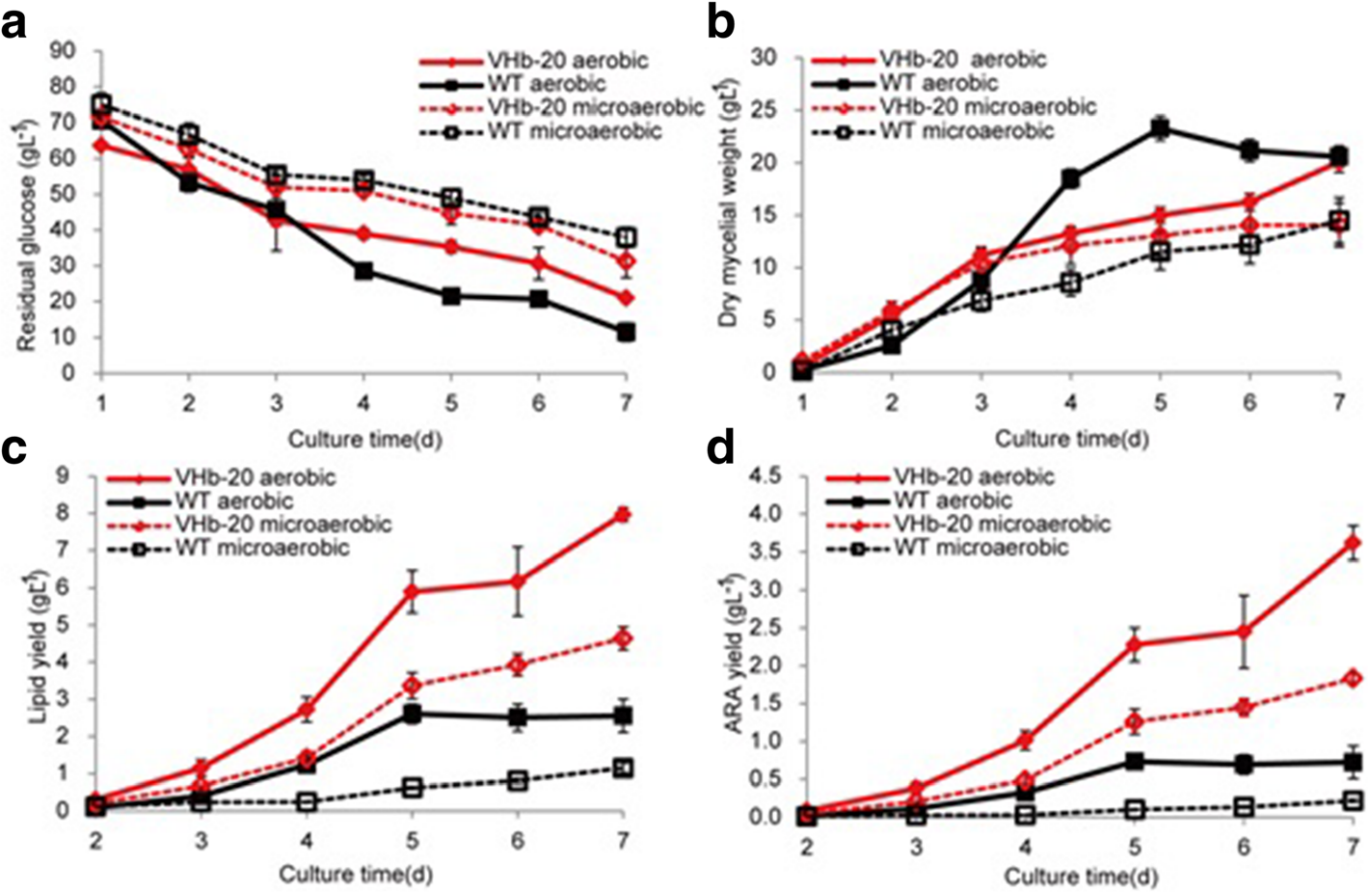
Comparison of growth profiles in shake flasks under aerobic and microaerobic conditions. **a** Residual glucose; **b** Dry mycelial weight; **c** Total lipid content; (**d**) Arachidonic acid production; The data are the mean of three repeats

Under aerobic conditions (cultivated in a 250-mL baffled flask containing 50 mL of medium at 25 °C and 200 rpm), the glucose consumption rates and dry weight of VHb-20 and wild-type strains were not obviously different after cultivation for 7 days (Fig. 2a and b), but the total lipid yield of strain VHb-20 (7.97 g L^−1^) was 3.15-fold higher than that of the wild-type strain (2.53 g L^−1^; Fig. 2c). In addition, the ARA production of the strain VHb-20 was 3.62 g L^−1^, which was 5.10 times higher than that of the wild-type strain (0.71 g L^−1^; Fig. 2d). To obtain a microaerobic condition with a lower level of dissolved oxygen, the liquid medium in the flask was increased from 50 mL to 150 mL. Under these conditions, the VHb-20 strain had a higher glucose consumption rate and growth rate compared with that of the control strain (Fig. 2a and b), and lipid and ARA production of VHb-20 was also higher by 4.03 folds (4.64 g L ^−1^ vs. 1.15 g L^−1^) and 8.36 folds (1.84 g L ^−1^ vs. 0.22 g L ^−1^), respectively (Fig. 2c, and d).

### Effect of VHb expression on the composition of fatty acids in *M. alpina*

**Fig. 3:**
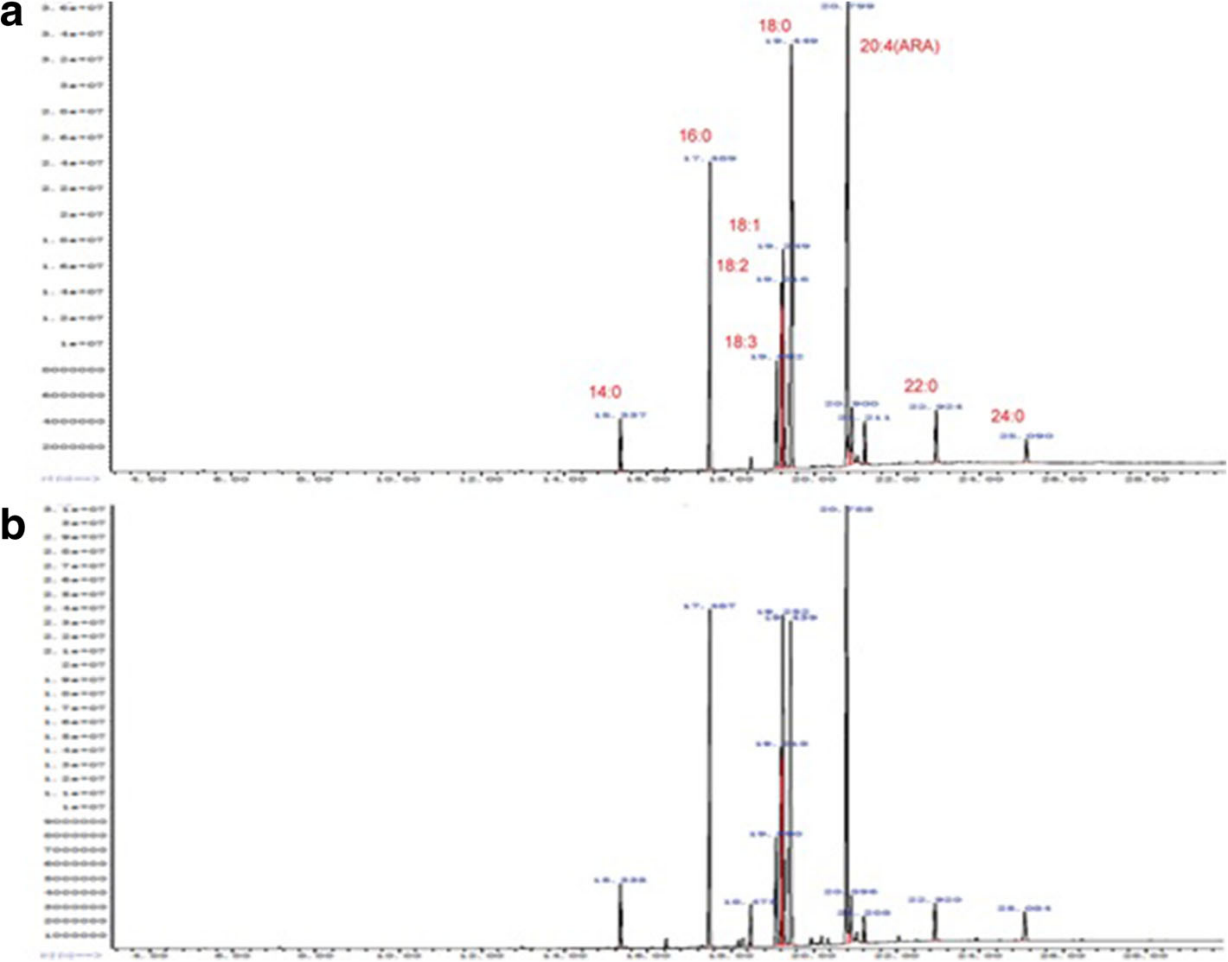
GC-MS analysis of fatty acids composition from wild type and VHb-20. **a** VHb-20 in shake flasks with free air diffusion; **b** Wild type in shake flasks with free air diffusion

The concentration of dissolved oxygen affects not only cell growth but also the synthesis of fatty acids. Therefore, we further compared the fatty acid composition and content of VHb-20 and the wild-type strain under aerobic or microaerobic conditions. VHb-20 and the wild-type strain produced similar types of fatty acids (Fig. 3), but the proportion of various fatty acids in the lipids had changed significantly (Table 1).The results showed that the proportion of palmitic acid (C16: 0), stearic acid (C18: 0) and oleic acid (C18: 1) in the total fatty acids of wild type strains was 15.82%, 17.98% and 17.58%, significantly higher than other fatty acids. Especially in the limited oxygen condition, the accumulation of oleic acid (C18: 1) is very huge, the content in the total fatty acids as high as 39.61%, resulting in ARA content was only 19.27%. However, the contents of palmitic acid (C16: 0) and oleic acid (C18: 1) in VHb-20 strain were significantly decreased, and the ARA content was increased to 45.68% and 38.51%, under the aerobic or microaerobic conditions respectively, which were nearly 1.7 folds and 2.0 folds as high as that of the control.

**Table 1:** Fatty Acid Compositions of wild type and VHb-20 in shake flasks

Fatty acid	Content (% total fatty acids)			
	WT(aerobic)	VHb-20(aerobic)	WT(microaerobic)	VHb-20(microaerobic)
14:0	3.22 ± 0.14	0.73 ± 0.02**	2.84 ± 0.04	1.46 ± 0.17**
16:0	15.82 ± 0.33	9.72 ± 0.36**	13.35 ± 0.10	11.51 ± 0.24**
18:0	17.98 ± 0.49	19.63 ± 0.73**	13.34 ± 0.95	23.19 ± 0.48**
18:1	17.58 ± 0.58	6.92 ± 0.55**	39.61 ± 2.26	8.34 ± 0.24**
18:2	7.43 ± 0.44	5.82 ± 0.22**	2.87 ± 0.35	5.94 ± 0.56**
18:3	3.37 ± 0.11	3.74 ± 0.08*	2.27 ± 0.38	3.71 ± 0.22*
20:3	3.43 ± 0.06	2.84 ± 0.19*	3.71 ± 0.08	2.38 ± 0.26**
20:4(ARA)	27.30 ± 0.77	45.68 ± 1.09**	19.27 ± 1.18	38.51 ± 0.44**

**p < 0.01,*p < 0.05

### Effects of VHb expression on the expression of desaturase genes

**Fig. 4:**
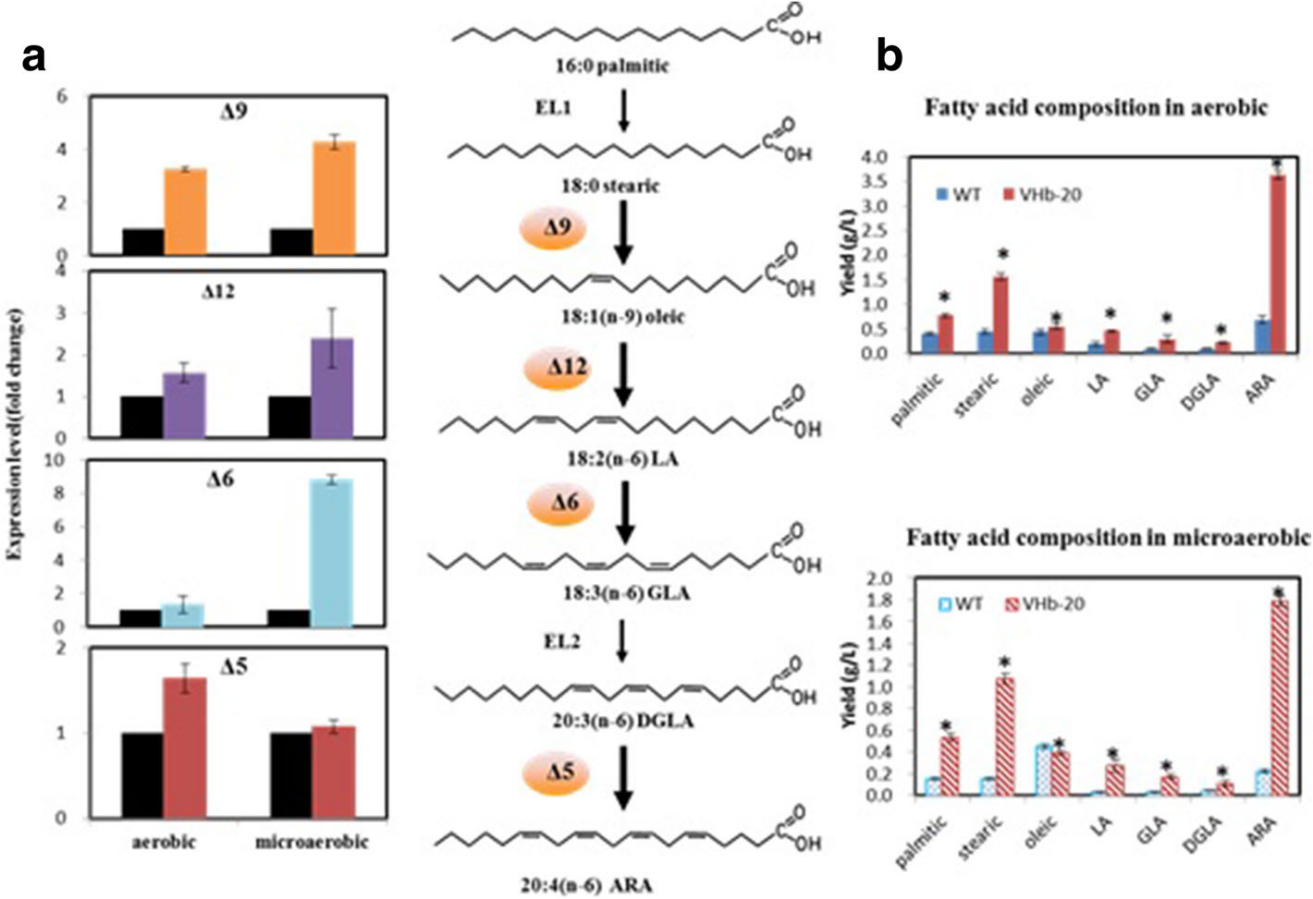
The changes of the expression of desaturases (Δ9 desaturase, Δ12 desaturase, Δ6 desaturase, Δ5 desaturase) and ARA production between the WT and VHb-20 strains under aerobic and microaerobic conditions. **a** Changes in the expression of desaturases, Black: WT; Colored: VHb-20 strain; **b** Changes in unsaturated fatty acid production (*p < 0.05)

The biosynthesis of unsaturated fatty acids in cell is catalyzed by desaturases, which require oxygen as a substrate [3]. Therefore, the expression of desaturase genes, including Δ9 desaturase, Δ12 desaturase, Δ6 desaturase, Δ5 desaturase, was investigated to verify the effects of VHb expression on the biosynthesis of unsaturated fatty acids at the molecular level. All the expression levels of these desaturase genes were higher in VHb-20, as compared with the wild type strain, in both aerobic and microaerobic conditions (Fig. 4a). From the results, Δ9 desaturase, which is the first enzyme in unsaturated fatty acid biosynthesis, was most sensitive to the expression of VHb, which resulted 3.2 and 4.2-fold higher expression of the Δ9 desaturase gene under aerobic and microaerobic conditions, respectively. A lesser effect was observed in the increased expression of Δ12 and Δ6 desaturase, which were only 1.4-fold higher in VHb-20, and 1.6-fold higher expression of Δ5 desaturase was observed under aerobic conditions. However, under microaerobic conditions, the expression level of Δ12 and Δ6 desaturase was increased by 2.3 and 8.7 times higher than that of the control strain. In agreement with the differences in the levels of these desaturases, the biosynthesis of unsaturated fatty acids was augmented, and the ARA production of VHb-20 was increased to 3.62 g L^−1^ and 1.84 g L ^−1^ under the aerobic and microaerobic conditions (Fig. 4b), respectively.

### Confirmation of the effects of VHb expression on cell growth and ARA production by using bioreactor fermentation

**Fig. 5:**
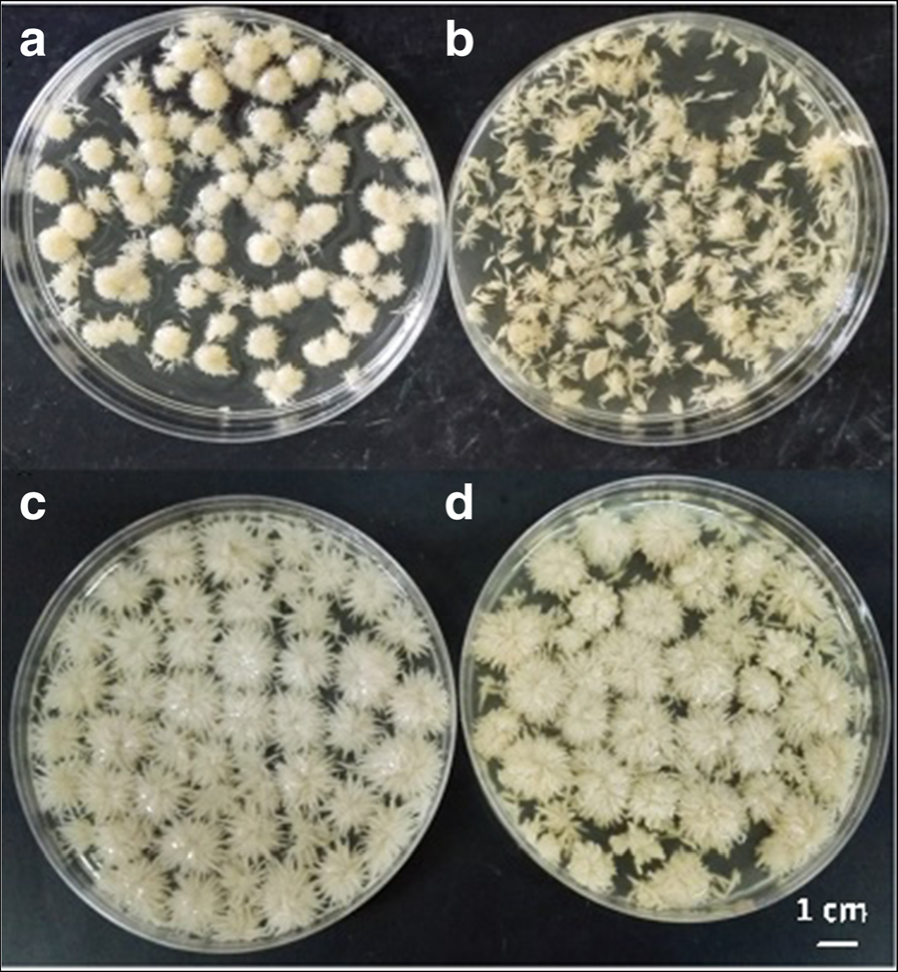
Morphology of hyphae in 5 L fermenter. **a** VHb-20 transformant under aerobic conditions; **b** wild type under vaerobic conditions; **c** VHb-20 transformant under microaerobic conditions; **d** wild type under microaerobic conditions

**Fig. 6:**
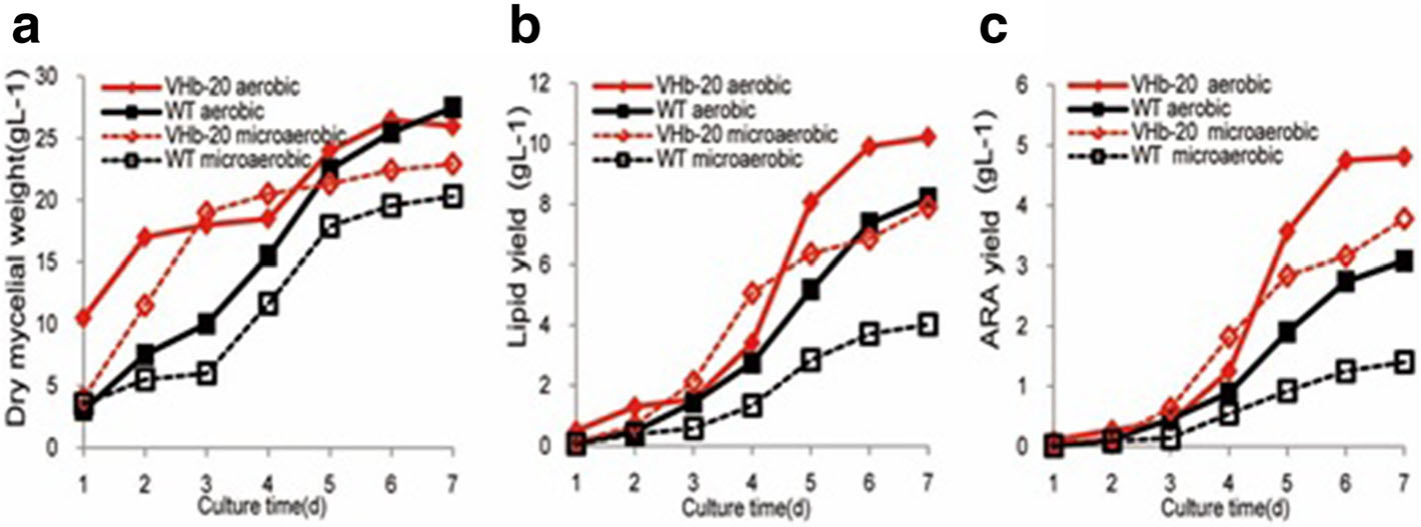
Comparison of growth profiles in a 5-L fermenter under aerobic and microaerobic conditions. **a** Dry mycelial weight; **b** Total lipid content; **c** Arachidonic acid production

Bioreactor cultures were grown to confirm the influence of VHb expression on cell growth and lipid production under both non-limiting (1.7vvm, 200 rpm) and limiting (0.8vvm, 100 rpm) dO2 conditions. From a morphological viewpoint (Fig. 5), VHb-20 grew faster and formed more uniform mycelial pellets than did the wild-type strain *M. alpina* ATCC 32222. In hypoxic conditions, the VHb-20 strain grew faster than the wild-type strain and reached the maximum biomass (12.90% more than the wild type) sooner (Fig. 6a). In addition, the culture of the wild-type strain clumped heavily and became difficult to stir after 3 days of cultivation, whereas uniform mycelial pellets were observed in the VHb-20 culture, and the culture broth maintained good fluid properties through 7 days of fermentation.

In agreement with the results of the shake flask assay, VHb expression also enhanced lipid and ARA biosynthesis in the bioreactor experiments, especially under limiting oxygen conditions. There was almost no difference in the lipid content of the DCW of VHb-20 in the two conditions (39.30% vs 34.40% for the normal and limiting oxygen conditions, respectively), but in the control, the lipid content was 34% lower in limiting oxygen conditions (29.80% vs 19.80%) at the end of fermentation. The maximum lipid yield of VHb-20 strain was 10.21 g L^−1^ and 7.89 g L ^−1^, which was nearly 1.25 and 1.96 fold higher than that of the original strain under the two aerobic conditions (Fig. 6b).

Under aerobic conditions, VHb-20 had a higher ARA yield by 1.56-fold, from a value of 3.08 g L^−1^ to 4.81 g L^−1^, compared with the original strain *M. alpina* ATCC 32222. At the end of fermentation, the ARA yield of VHb-20 was 2.70 times (3.78 g L^−1^ vs 1.40 g L^−1^) higher than that of the wild type under microaerobic conditions (Fig. 6c).

## Discussion

ARA is an essential polyunsaturated fatty acid in human nutrition that has broad applications in many industries [26]. Although *M. alpina* is considered to be a good production strain for high level of ARA production, the low percentage of ARA in total lipids results in a production bottleneck [27]. In this study, we demonstrated that expression of *Vitreoscilla* hemoglobin significantly improves ARA and total lipid production in *M. alpina*.

Heterologous expression of VHb in *M. alpina* increased cell growth as well as oxygen uptake under non-limiting and limiting dO2 conditions. Furthermore, VHb expression shortened the lag phase for the growth of *M. alpina* and the appearance of ARA. A similar observation has previously been reported in *S. occidentalis*, in which alpha-amylase appears earlier in the culture medium of VHb-expressing cells [28], and the result is also consistent with the expression of VHb in *Gluconobacter oxydans* [29] and *Phellinus igniarius* [8]*.* Importantly, the morphology of the VHb-20 strain changed significantly. The mutant VHb-20 stain formed small homogeneous globules, whereas the wild-type strain formed loose mycelia (Fig. 5). Previous experiments have shown that in late fermentation stages, the wild-type strain readily agglomerates, thus resulting in decreased dissolved oxygen. In contrast, mycelial pellets increased the fermentation fluidity, which is more conducive to dissolved oxygen and accumulation of lipids and ARA [7]. Moreover, the growth pattern of *M. alpina* has a large effect on ARA production [30]. These results are also consistent with findings by Higashiyama et al. [31] indicating that high concentrations of dissolved oxygen are necessary for obtaining smooth pellets, and the number of spherical fluffy pellets and the dissolved oxygen concentration are positively correlated.

Dissolved oxygen levels affect not only cell growth but also the synthesis of fatty acids in *M. alpina*. Therefore, the VHb-20 and wild-type strains were analyzed for fatty acid and ARA content under normal culture conditions and dissolved oxygen-limiting conditions. Studies have shown that the expression of VHb increases the yield of ARA and total lipids, especially under oxygen-limiting conditions. It has been reported previously that VHb enhances ATP production and respiratory activity [32]. Evidence has shown that VHb improves the efficiency of oxidative phosphorylation of cells by regulating the activity of the relevant respiratory oxidase, thereby changing the central carbon metabolic pathway, and ultimately promoting cell growth and protein expression under hypoxic conditions [33, 34]. In addition, VHb also has peroxidase activity, and many heterologous hosts protect against oxidative invasion by expressing this gene, in agreement with the hypothesis that VHb is a carrier of oxygen in the cell [12, 35]. On the basis of these observations, VHb expression may promote fatty acid synthesis and increase total lipid accumulation, owing to the enhancement of respiratory activity and ATP supply.

In addition, another prominent finding was that VHb expression significantly changed the fatty acid composition, particularly by decreasing the ratio of C16:0 and C18:1 and increasing the ratio of C20:4 (ARA; Table 1). The biosynthesis of ARA strongly depends on oxygen accessibility, and some specific catalytic functions of various fatty acid desaturases and elongation enzymes involved in ARA synthesis in *M. alpina* have been elucidated [36, 37]. The oxygen requirement for desaturation was higher than that for cell growth and total lipid production. Therefore, the increase in ARA content in VHb-20 may be a result of the overall increase of the fatty acid desaturase caused by the presence of heterologous hemoglobin. This speculation is consistent with the expression level of desaturases that we determined by qRT-PCR. It can be seen from Fig. 4 that four kinds of desaturases in the ARA metabolic pathway are Δ9-, Δ12-, Δ6-, Δ5- desaturases respectively, and their expression levels are significantly up-regulated under both the aerobic and microaerobic conditions. Moreover, these desaturases were the key enzymes catalyzed the palmitic acid (C16: 0), stearic acid (C18: 0) and Oleic acid (C18: 1) converted to ARA. Therefore, we hypothesized that the increase of ARA production of *M. alpina* is probably due to the increase of the expression of desaturase and the improved desaturase activities in ARA metabolic pathway and the promotion of metabolic flow to ARA synthesis. More detailed mechanisms need further study.

## Conclusions

In conclusion, the successful expression of VHb in *M. alpina* significantly increases ARA and lipid production as well as promotes cell growth and increase viability under both lower and normal dissolved oxygen conditions. Therefore, we suggest that expression of the *vgb* gene in *M. alpina* might have good prospects in high-density fermentation. However, the specific mechanisms underlying the effects of VHb in *M. alpina* are not clear and require further investigation.

## Additional file

Additional file 1: (DOCX 263 kb)

## Abbreviations

ARA: Arachidonic acid
ATMT: *Agrobacterium tumefaciens-*mediated transformation
CO: Carbon monoxide
DCW: Dry cell weight
DGLA: Dihomo-γ-linolenic acid
DO: Dissolved oxygen
FAMEs: Fatty acid methyl esters
FID: Flame ionization detector
GLA: γ-linolenic acid
HPH: hygromycinb phosphotransferase gene
IM: Inducing medium
LA: Linoleic acid
*M. alpina*: *Mortierella alpina*
MM: Minimal medium
PUFAs: Polyunsaturated fatty acids
VHb: *Vitreoscilla* hemoglobin
