# Supplementary material for: Expression of Vitreoscilla hemoglobin enhances production of arachidonic acid and lipids in Mortierella alpina
Source: BMC Biotechnol. 2017 Aug 30;17:68. doi: 10.1186/s12896-017-0388-8 (PMC5577678; doi:10.1186/s12896-017-0388-8)

# Expression of *Vitreoscilla* hemoglobin enhances production of arachidonic acid and lipids in *Mortierella alpina*

Huidan Zhang^1,3,4^, Yingang Feng^1,3^, Qiu Cui^1,2,3^*, Xiaojin Song^1,3,^*

1 Shandong Provincial Key Laboratory of Energy Genetics, Qingdao Institute of Bioenergy and Bioprocess Technology, Chinese Academy of Sciences, Qingdao 266101, Shandong, China

2 Key Laboratory of Biofuels, Qingdao Institute of Bioenergy and Bioprocess Technology, Chinese Academy of Sciences, Qingdao 266101, Shandong, China

3 Qingdao Engineering Laboratory of Single Cell Oil, Qingdao 266101, Shandong, China

4 University of Chinese Academy of Sciences, Beijing 100049, China

**Corresponding Author:**

**Name:** Xiaojin Song

**Post Address:** No.189 Songling Road, Laoshan District, Qingdao 266101, Shandong Province, China

**Tel.:**+86 532 80662705; **Fax:** +86 532 80662707;

**E-mail:** [songxj@qibebt.ac.cn](mailto:songxj@qibebt.ac.cn).

**Name:** Qiu Cui

**Post Address:** No.189 Songling Road, Laoshan District, Qingdao 266101, Shandong Province, China

# Supporting information

Table S1. Strain and plasmid used in this study.

| Strain or plasmid | Description and relevant characteristics | Source or reference |
| --- | --- | --- |
| Strain |  |  |
| *Mortierella alpina*  ATCC 32222 | \| Wild-type *Mortierella alpina* strain \|  \| \| --- \| --- \| | American Type Culture Collection |
| VHb-20 | Derived from ATCC 32222, containing *CBXB* and *vgb* expression cassette | This study |
| *E. coli* |  |  |
| DH5α | Cloning strain | Invitrogen |
| Plasmid |  |  |
| pMD19-T | *amp^R^,* cloning vector | Takara |
| pUC57-vgb | *amp^R^,*containg optimized *vgb* gene sequences | GenScript |
| pMD19T-HPH | *amp^R^,* containg HPH expression cassette | This study |
| pMD19T-HPH-18S | *amp^R^,* containg HPH expression cassette and 18S rDNA homologous arm | This study |
| pMD19T-CBXB-18S | *CBXB^R^*, containg *CBXB* expression cassette, hisH4.1 promotor  and trpC terminator | This study |
| pBIG4MRHrev | *kan^R^*, containg HPH expression cassette | provided by Yasuyuki Kubo |
| pBIG-CBXB | *kan^R^*, *CBXB^R^*, containg *CBXB* expression cassette, hisH4.1 promotor and trpC terminator | This study |
| pBIG-CBXB-VHb | *kan^R^*, *CBXB^R^*, containg *CBXB* and *vgb* expression cassette, hisH4.1 promotor and trpC terminator | This study |

Table S2 Oligonucleotides used in vector construction.

| Primers | Sequence (5′-3′) | Note |
| --- | --- | --- |
| P1 | AAGCGAAAGAGAGATATGAAACA | To amplify HPH expression cassette |
| T1 | GTAAACGACTCATAGGAGAGTTGT |  |
| 18S-F | TGCTCTAGACAATTGGAGGGCAAGTCTGG | To amplify 18S rRNA sequence |
| 18S-R | TCCCCCGGGTCAGTGTAGCGCGCGTGCGG |  |
| CBXB-F | GCTACATGTCAATGTCCCTCTCTATTGCCAAGCAGT | To amplify *CBXB* sequence |
| CBXB-R | CGCGGATCC TTACTCCAAA GCCATGGTCT TCTTGA |  |
| P2 | CCATCGATAAGCGAAAGAGAGATATGAAACA | To amplify *CBXB* expression cassette |
| T2 | CCGGAATTCGTAAACGACTCATAGGAGAGTTGT |  |
| P3-F | AAGCGAAAGAGAGATATGAAACA | To amplify histone H4.1 promoter |
| P3-R | GTTGATGGTC TGCTGGTCCA ACAT ATTGTTGAGA GAGTGTTGGG TGA |  |
| VHb-F | ATGTTGGACCAGCAGACCATCAAC | To amplify *vgb* gene |
| VHb-R | TTACTCGACG GCCTGGGCGT ACAAG |  |
| T4-F | CTTGTACGCC CAGGCCGTCG AGTAA TGAAATCATC AAACAGCTTG ACG | To amplify trpC terminator |
| T4-R | GTAAACGACTCATAGGAGAGTTGT |  |
| P5 | CCGGAATTCAAGCGAAAGAGAGATATGAAACA | To amplify *vgb* expression cassette |
| T5 | TCCCCCGGGGTAAACGACTCATAGGAGAGTTGT |  |

Table S3 Primers used in the qRT-PCR validation

| **Primers** | **Sequence(5’-3’)** | **Description** |
| --- | --- | --- |
| Δ5-RTF | TGTCTGGAAGATTCTGGGAGC |  |
| Δ5-RTR | TTTGGTTGGGCTTGATACGAC | Δ5 RT-qPCR |
| Δ6-RTF | TGTTCTGGCAGCAGTGCGGATGG |  |
| Δ6-RTR | GGCGTGGTGAGTGTTGTGCTTGTCC | Δ6 RT-qPCR |
| Δ9-RTF | TCGTTCAGTGGCAGCACAAGA |  |
| Δ9-RTR | CAAGACGGAGGATAGCAGCATA | Δ9 RT-qPCR |
| Δ12-RTF | TGGGTGCTGGCTCACGAGTGT |  |
| Δ12-RTR | CCAGTGGCCTTGTGGTGCTTC | Δ12 RT-qPCR |
| 18S-RTF | CGTACTACCGATTGAATGGCTTAG |  |
| 18S-RTR | CCTACGGAAACCTTGTTACGACT | Internal control for RT-qPCR |

Table S4. The sensitivity of *Mortierella alpina* ATCC 32222 to various antibiotics.

| **Antibiotics (μg/mL)** | **0** | **10** | **20** | **50** | **100** | **200** | **300** | **500** | **1000** | **2000** |
| --- | --- | --- | --- | --- | --- | --- | --- | --- | --- | --- |
| Carboxin | **+** | **+** | **+** | **+** | **±** | **-** | **­-** | **-** | **-** | **-** |
| Benomyl | **+** | **+** | **+** | **+** | **+** | **+** | **+** | **±** | **-** | **-** |
| Chloramphenicol | **+** | **+** | **+** | **+** | **+** | **+** | **+** | **+** | **±** | **-** |
| Neomycin (G418) | **+** | **+** | **+** | **+** | **+** | **+** | **+** | **+** | **+** | **±** |
| Zeocin | + | + | + | + | + | + | + | + | + | + |
| Hygromycin | + | + | + | + | + | + | + | + | + | + |
| Oligomycin | + | + | + | + | + | + | + | + | + | + |
| Phleomycin | + | + | + | + | + | + | + | + | + | + |
| Glufosinate | + | + | + | + | + | + | + | + | + | + |

**Figures**

Figure S1.Optimized *vgb* Sequence (Optimized Sequence Length:441bp, GC%:60.27)

ATGTTGGACCAGCAGACCATCAACATCATCAAGGCTACCGTCCCTGTCCTGAAGGAGCACGGCGTCACCATCACC

ACCACCTTCTACAAGAACCTCTTCGCTAAGCACCCTGAGGTCCGTCCTCTCTTCGACATGGGTCGTCAGGAGTCG

TTGGAGCAGCCCAAGGCTTTGGCTATGACCGTCCTGGCTGCTGCCCAGAACATCGAGAACCTGCCTGCTATCCTC

CCTGCTGTCAAGAAGATCGCCGTCAAGCACTGCCAGGCTGGTGTCGCTGCTGCTCACTACCCCATCGTCGGACAG

GAGCTCTTGGGCGCCATCAAGGAGGTCCTGGGAGACGCTGCTACCGACGACATCCTCGACGCTTGGGGCAAGGCC

TACGGTGTCATCGCCGACGTCTTCATCCAGGTCGAGGCCGACTTGTACGCCCAGGCCGTCGAGTAA

Figure S2. Schematic representation of the plasmid construction. his H4.1p, hisH4.1 promoter; *trpCt*, trpC terminator; *CBXB*, carboxin resistance gene; *RB* ,right border; *LB*,left border; *vgb*, optimized *vgb* gene.


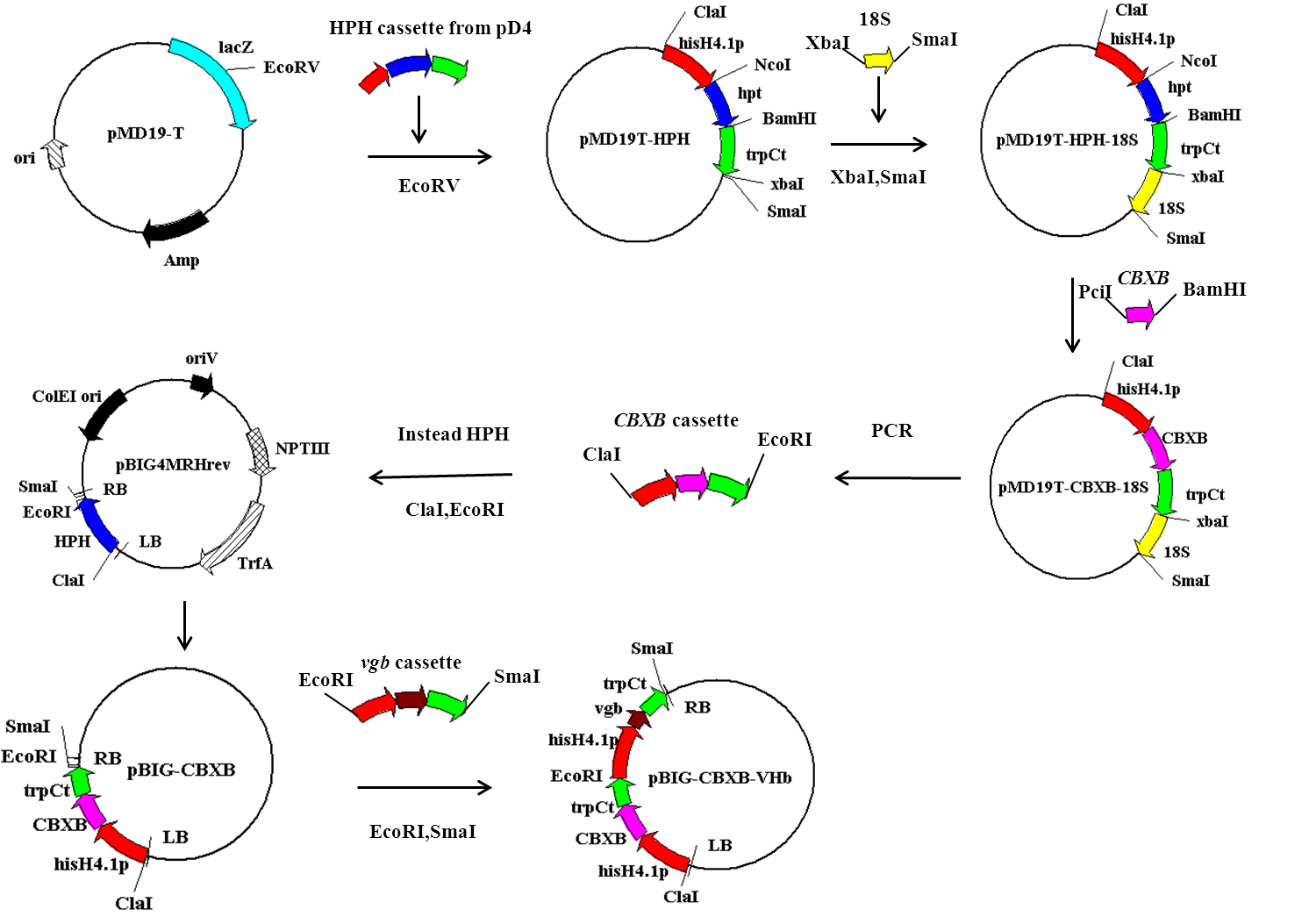

Supplement: Additional file 1: — (DOCX 263 kb) [file 12896_2017_388_MOESM1_ESM.docx]
